# Effectiveness of Concrete Reinforcement with Recycled Tyre Steel Fibres

**DOI:** 10.3390/ma15072444

**Published:** 2022-03-26

**Authors:** Agnieszka Michalik, Filip Chyliński, Jan Bobrowicz, Waldemar Pichór

**Affiliations:** 1 Building Structures, Geotechnics and Concrete Department, Instytut Techniki Budowlanej, ul. Filtrowa 1, 00-611 Warsaw, Poland; f.chylinski@itb.pl; 2 Certification Department, Instytut Techniki Budowlanej, ul. Filtrowa 1, 00-611 Warsaw, Poland; j.bobrowicz@itb.pl; 3 Department of Building Materials Technology, Faculty of Materials Science and Ceramics, AGH University of Science and Technology, al. Mickiewicza 30, 30-059 Cracow, Poland; pichor@agh.edu.pl

**Keywords:** recycled tyre steel fibres (RTSFs), manufactured steel fibres (MSFs), fibre-reinforced concrete (FRC), residual flexural tensile strength, crack mouth opening displacement (CMOD), deflection, work of fracture, toughness index I_5_, I_10_, I_20_, carbon footprint, sustainable construction

## Abstract

The role of searching for industrial waste management solutions in construction is key for environmental protection. Research in recent years has focused on solutions aimed at reducing the carbon footprint. This paper presents the results of tests conducted on concrete reinforced with treated recycled tyre steel fibres (RTSFs) compared to the same amount of manufactured steel fibres (MSFs). The effectiveness of concrete reinforcement with RTSFs was analysed using the fracture mechanics parameters of cementitious composites. Rheological tests, residual flexural tensile strength tests, work of fracture measurements, toughness indices, examinations of the fibre distribution in the concrete, and SEM observations of the concrete fractures with fibres were performed. Determining the work of fracture and toughness indices was an innovative aspect of this paper. As the amount of RTSFs increased, a decrease in the consistency was observed, although the distribution of fibres in the concrete was uniform, as proven by the results of computer tomography tests. Concrete reinforced with RTSFs that is purified and refined during the recycling process might have better properties than concrete reinforced with the same amount of MSFs. The application of RTSFs in construction has environmental and economic benefits in addition to the strengthening of cementitious composites.

## 1. Introduction

The dynamic development of transport infrastructure and the automotive industry has forced an increase in the number of vehicles and, thus, an increase in the production of rubber tyres, which when worn out pollute the environment. The disposal of tyres is a global environmental problem. About 1.5 billion tyres are produced annually worldwide [1,2]. More than 500 million used tyres are stored in landfills [3] and pose serious threats to humans and the environment [4]. They might cause fires that are difficult to extinguish and contaminate soil, groundwater, and specific flora and fauna that create the conditions for epidemic diseases [5].

Climate change, environmental degradation, and an increasing amount of waste pose a threat to Europe and the rest of the world. In order to address these challenges, the European Commission has introduced a strategy called the European Green Deal [6], which includes an action plan that facilitates, among other things, the more efficient use of resources through the transition to a circular economy, counteracting the loss of biodiversity and reducing pollution levels. Considering the above, it is necessary to save natural resources as much as possible and reuse industrial waste.

Tyres are a type of waste that does not decompose naturally and must undergo one form of recycling [7]. Apart from rubber, used car tyres contain textile and steel cords which make them very difficult to recycle. Vehicle tyre disposal methods can be divided into three main groups: product recycling, energy recycling, and material recycling. Product recycling refers to the use of used tyres in their entirety, whereby their durability and ability to absorb noise, shocks, and impacts is exploited. Energy recycling involves recovering energy, usually by burning the tyres, whereby the raw material is converted into heat or electricity. Energy is obtained by burning all or part of the tyres in specially adapted furnaces, mainly in cement plants and also in district heating stations. Material recycling is the reuse of the raw materials of a tyre. The basic process for the material recycling of waste tyres is shredding and separation into separate material groups: rubber, textile cords, and steel cords.

According to statistics published by ETRMA [8], in EU countries in 2018, 91% of waste tyres were further processed. Material recycling in EU countries constituted 62% and energy recycling 38%. Energy recycling, however, is not the best way to dispose of tyres, as the production of tyre rubber consumes several times more energy compared to the energy recovered by energy recycling [7]. Consequently, the use of recycled rubber for its original purpose rather than incineration make more sense, both economically and environmentally. Therefore, taking into account the principles of sustainable construction and the European Green Deal, material recycling, i.e., the reuse of the rubber, textiles, and steel obtained from the tyre, is the most beneficial for the environment.

The use of secondary raw materials from the material recycling of tyres has been the subject of much research for many years. The recycled material that has proved to have the most widespread application is rubber waste [9], constituting the so-called rubber aggregate of various fractions, which is used as a material for the base of roads, asphalt surfaces, embankments, playgrounds, noise barriers, and as an aggregate for concretes and mortars [3,10,11,12,13,14]. Additionally, some scientific papers focus on the use of recycled tyre steel cords as structural reinforcement for concrete, either as a standalone reinforcement or as a mix of different types of fibres [1,2,15,16,17,18,19].

The search for a way to replace traditional steel fibre reinforcement in concrete is crucial for environmental protection for a number of reasons. The world is facing a threat of a shortage of natural resources, including raw materials for steel production; thus, conserving natural resources is being emphasised. In addition, the steel production process contributes significantly to the increase in greenhouse gases polluting the atmosphere—steel production generates a significant amount of greenhouse gases; the carbon footprint of steel production is 1900 kg eCO_2_/t [20]. Steel is one of the main components of a tyre (approx. 13–27%) [5], which means that the efficient use of recycled steel materials from tyres can significantly mitigate the problems caused by waste tyres. Recycling used tyres can stop about 1524 tonnes of CO_2_ emissions per year. Every year, about 3.4 million tonnes of old tyres are disposed of in Europe [21,22]. It has been scientifically proven that the material recycling process for rubber tyres, consisting of the separation of steel from rubber and textiles through cutting, air, and magnetic separation, as well as an additional treating of the steel cord, is more environmentally beneficial than the production of new steel [21,23,24,25].

In addition, numerous studies indicate that steel cord is an effective material for reinforcement, comparable to manufactured steel fibres. Over the past few years, one could spot the increasing interest of researchers in the use of recycled steel fibres from tyres as structural reinforcement for concrete and as a possible replacement for manufactured steel fibres. A literature analysis indicates that recycled tyre fibres were more effective in improving the flexural strength of concrete and improving its fracture properties compared to unreinforced concrete and concrete with steel fibre additions, provided that they were found in the appropriate quantity and purity in concrete [1,2,26,27,28,29,30,31,32,33].

Studies of the effects of various types of tyre fibres and their mixture with manufactured steel fibres on the rheological and mechanical properties of concrete [26,34,35,36] indicate that, due to the presence of rubber and textile contaminants and steel dust, higher amounts of the steel cord obtained from tyres should be added compared to manufactured steel fibres. The most commonly used and financially viable tyre recycling methods involve a combination of mechanical shredding and granulation to produce steel fibres with irregular shapes, lengths, and diameters. These fibres, however, are often heavily contaminated with rubber (up to 20% by weight) [15]. Therefore, further processing is required to minimise rubber contamination to less than 0.5% by weight [15] and limit fibre length and diameter distribution to achieve values that are effective in concrete whilst ensuring good fibre homogenisation during concrete mixing. Only after treating and sorting can RTSFs be used in concrete as a structural reinforcement [2,15,24,37,38].

Contaminants from rubber and textile cord as well as steel dust encase the steel fibres and reduce the effectiveness of the reinforcement. The authors of [39] investigated the effect of the length and cleanliness of recycled tyre steel fibres (RTSFs) on the flexural tensile strength of ultra-high-performance concrete and showed that rubber and other contaminants, as well as fibres less than 9 mm in length, significantly reduced the residual flexural tensile strengths of the concrete. The treating and reduction of short fibres are necessary to obtain better mechanical properties of cement composites.

In one paper [15], the mechanical properties of concrete beams with the addition of treated blended manufactured steel fibres (MSFs) and recycled tyre steel fibres (RTSFs) were investigated. The residual flexural tensile strength and elastic modulus were investigated. In this study, treated tyre steel fibres and two types of manufactured corrugated steel fibres were tested. The fibre contents were 30, 35, and 45 kg/m^3^, respectively. The results indicated that the treated RTSFs were more effective in controlling microcracking than the originally obtained steel cord.

Despite the good scientific evidence, there are still concerns about the use of RTSFs for concrete reinforcement. The reason for the lack of interest in the application of tyre steel fibres as concrete reinforcement is related to their geometric parameters. RTSFs are very different from all other manufactured steel fibres on the market and are commonly used as structural reinforcement in concrete structures, such as industrial floors and tunnel linings [40,41]. As they are obtained from the recycling of used tyres, their parameters are dependent on the processing technology used, and it is impossible to control their dimensions and geometry. It is well known that the factors that have the greatest influence on the effectiveness of fibre reinforcement in a cement matrix are the fibres’ geometric parameters, such as their length, diameter, and shape [42].

Considering the geometry, hybrid fibres such as those from recycled tyres, i.e., blended fibres of different lengths, diameters, and shapes, can be more effective in terms of reinforcement than dimensionally homogeneous manufactured steel fibres, a fact which has been scientifically proven [43]. The use of homogeneous fibres can be effective in preventing the cracking of a certain width, but the cement matrix crack process is more complicated and gradual [44]. The use of blended fibres with different aspect ratios (length/diameter) and physical properties can provide better crack control over a wider range of crack widths. The process of crack bridging by fibres can take place on different planes of the brittle cement matrix. Numerous studies have shown that using hybrid fibres can ensure better performance than using single-type fibres [45,46,47,48,49,50,51].

Taking into account the above considerations, the attempt to use treated recycled tyre steel fibres as structural reinforcement for concrete, from the point of view of the conservation of natural resources, waste management, and the reduction of the carbon footprint, while effectively reinforcing the cement matrix, is justified and fits in with the ideas shaping the innovation challenges of construction technology [52].

The purpose of this study is to analyse the effectiveness of concrete reinforcement with recycled tyre steel fibres that have been cleansed of rubber and textile contaminants. As part of the research, a comparative study on concretes with the addition of treated tyre steel fibres and concretes with the same amount of manufactured steel hooked-end fibres, commonly used as structural reinforcement for concrete, was performed. Rheological tests of the concrete mixture (consistency) and the concretes with the same amount of RTSFs as MSFs were performed. As a measure of the effectiveness of the reinforcement, residual flexural tensile strength tests and tests defining the fracture toughness were performed, i.e., work of fracture and fracture toughness indices.

In this study, the fibre reinforcement effectiveness was determined by examining the works of fracture; no similar research studies were found in the literature. The reinforcement effectiveness measure was also determined by a fracture’s toughness indices. Moreover, the novelty of this paper is that the properties of concrete with purified RTSFs were compared with the properties of concrete containing the same amount (by mass) of industrial fibres used as scattered reinforcement for MSF concrete. In similar studies, RTSFs were typically added in higher quantities than MSFs. Recycled tyre fibre treatment technology contributes to their better adhesion and effective concrete reinforcement. The fibre purity grade was evaluated with microscopic analysis, while a modern 3D computer tomography method was used for evaluating the fibre distribution in the concrete. 

## 2. Materials and Methods

### 2.1. Materials

Two types of fibre were used in these tests: manufactured steel fibre (MSF) and recycled tyre steel fibre (RTSF). The characteristics of the fibres are presented in Table 1. The dimensions of all fibers were tested according to EN 14889-1 [53] on 30 randomly selected fibres.

**Figure 1:**
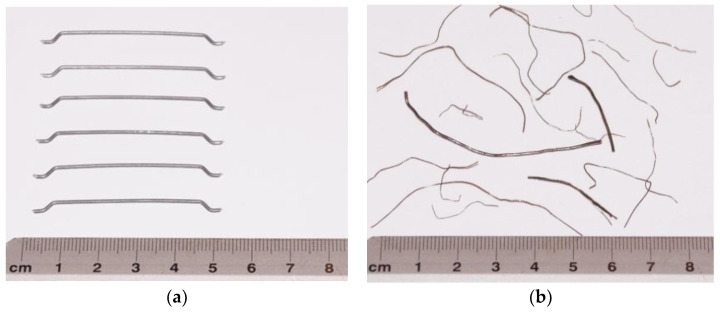
Pictures of steel fibres: (**a**) MSFs, (**b**) RTSFs.

MSFs are used as structural reinforcement for concrete and meet the requirements of the standard [53]. The type of material declared by the manufacturer was SAE 1006: round wire, cold-drawn, bare, with a nominal diameter of 1.0 mm, and made of low-carbon steel (below 0.1% C). RTSFs were obtained by means of the mechanical recycling of tyres and had undergone additional treatment. Additional treatment was based on the use of air, vibration, and magnetic separators, and the final separation of textile remnants took place using negative pressure. After separation, a fraction of rubber granulate and steel cord was obtained. Pictures of RTSFs and MSFs used in this study are shown in Figure 1.

**Figure 2:**
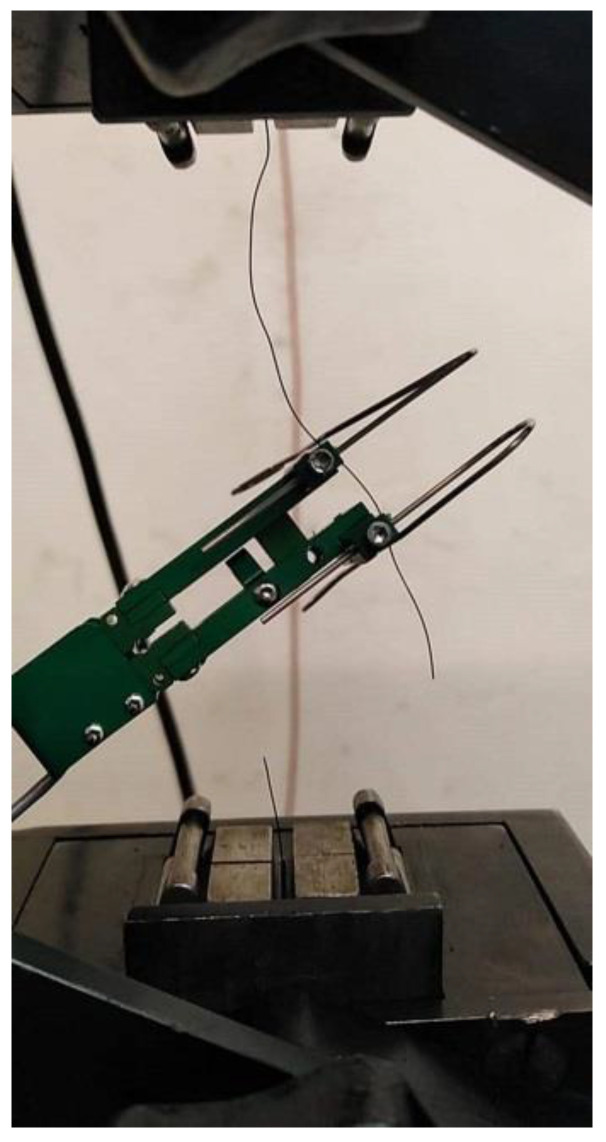
Broken wire from tyre in a breaking test.

Table 2 shows the comparison of tensile strength and elastic modulus according to EN ISO 6892-1 [54]. The test, for both RTSFs and MSFs, was conducted on thirty wires approx. 30–40 cm in length. Figure 2 shows the measurements of the tensile strength and modulus of elasticity of wires from tyres. An extensometer was mounted on the wire. The wires obtained from the tyres received the demanded length and, due to the variety of diameters, were divided into two groups; 30 wires were tested in each group.

The results of the study indicated that fibres (wires) from tyres, despite their years of use and the process of recycling and pulling from tyres, show approx. 30–50% higher tensile strength compared to manufactured steel fibres, demonstrating great potential for their future application. Literature data indicate that the steel matrix in the tyre, which is the structural frame on which all loads are applied, is produced from high-grade and high-strength steel, with an average tensile strength of 2500 MPa [15,39]. Wires obtained from recycled tyres showed lower values of elastic modulus than manufactured wires, with values being lower for smaller wire diameters. The data presented in this study are available in the Supplementary Material.

### 2.2. Methods

#### 2.2.1. Composition of Concrete with Fibres

In this study, concretes with the addition of manufactured steel fibres and the same amount of recycled tyre steel fibres were tested in order to compare and evaluate the reinforcement efficiency of both types of fibre. The concrete was made according to EN 14845-1 [55]. Table 3 shows the composition of the reference concrete. The reference concrete, according to EN 14845-1 [55], should be designed as free of plasticising admixture to determine the fibres’ impact on the reference mix with no admixture impact on the consistency.

The composition of the selected reference concrete met the following requirements:

- Maximum content of cement CEM 42.5 R 350 kg/m^3^;
- Maximum aggregate size 16 mm, natural, uncrushed, silica-based;
- Water/cement ratio 0.55;
- Flexural tensile strength 4.3 ± 0.3 MPa (min. concrete class C25/30);
- Consistency of mix tested with the Vebe V3 method (from 6 to 10 s).

For the concrete made as stated in Table 3, laboratory tests were carried out with different amounts of structural reinforcement to determine the maximum amount of fibres to be added. Preliminary tests of the concrete mixes indicated that when the recycled tyre steel fibre content was higher than 40 kg/m^3^, there was an issue concerning the appropriate homogenisation in the concrete mix. In addition, a fibre content higher than 40 kg/m^3^ resulted in a decrease in the workability, while the reference concrete excluded the dispensing of a chemical admixture. For the tests, the concrete was made as stated in Table 2, with MSF and RTSF contents of 10, 20, 30, and 40 kg/m^3^. Eight concrete beams of 150 mm × 150 mm × 550 mm were made for each type of concrete, which, after demoulding, were cured for 28 days in water at 20 ± 2 °C. The tested concretes are shown in Table 4. A batch of about 200 kg of RTSFs obtained from the same production line was provided for testing. Before testing, the samples were homogenized and averaged. It can be concluded that the tested samples had the same distribution of length and diameter.

#### 2.2.2. Consistency of Concrete with Fibres

The consistency of concrete mixes with contents stated in Table 3 and Table 4 was tested using Vebe and the fall-cone method according to EN 12350-3 [56]. MSFs and RTSFs were added manually into the concrete mix and mixed for about 5–10 min to achieve the necessary fibre homogenisation.

#### 2.2.3. Residual Flexural Tensile Strength

**Figure 3:**
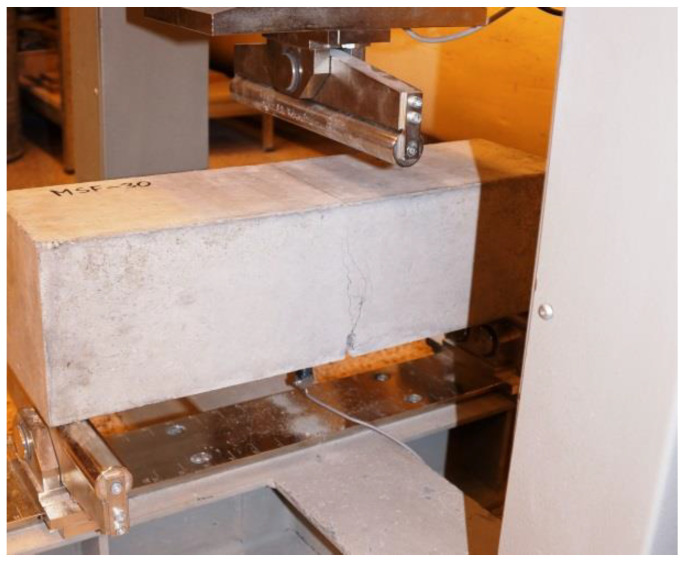
Three-point bending of a beam with a notch.

The residual flexural tensile strength after 28 days was tested according to EN 14651 [57], using a three-point bending with a notch. The test was performed on eight 150 mm × 150 mm × 550 mm specimens for each mix, with about a 4 mm wide and 25 mm deep notch in the middle of the beam for the purpose of testing the propagation of the beam crack’s location. Figure 3 shows the test rig for a random sample. The residual flexural tensile strength test result is the average of the eight specimens. During bending, the load (kN) and CMOD (crack mouth opening displacement (mm)) were read at a frequency of 5 Hz up to CMOD = 3.5 mm, allowing approximately 5200 load/CMOD results to be read. The load increment rate used was 0.05 mm/min up to CMOD = 0.1 mm, and then 0.2 mm/min. The crack occurred at the notch and propagated through the beam cross-section concerning all specimens tested. The first fracture (proportionality limit) was determined according to EN 14651 (58) as the highest load at the CMOD from 0 to 50 µm.

In most cases, both RTSF and MSF beams tended to break vertically along the notch and branch, as shown in Figure 3. No significant differences in fracture characteristics were observed with the amount of RTSF and MSF fibres.

#### 2.2.4. Work of Fracture

The work of fracture of fibre concrete was defined as the total area under the load–deflection curve. The load–deflection diagrams for each test specimen were converted from the load–CMOD curves using the deflection conversion factor δ = (0.85CMOD + 0.04) according to EN 14651 [57]. For each figure, the work of fracture was determined up to CMOD = 3.5 mm (deflection = 3.02 mm). The work of fracture result is the average of the eight specimens for each concrete mix with fibres.

In some papers, fracture resistance for fibre-reinforced composites was determined by the fracture energy [58,59,60], which, in addition to the area under the load–deflection curve, considers additional parameters such as dimensions, sample weight, and acceleration due to gravity. The authors state that the fracture energy defines a controlled crack growth during a fracture process caused by the force applied in the case of a strictly brittle fracture. In the case of fracture of cement composites containing fibres, the process occurring after the first crack in the cement matrix is made up of several processes, such as the removal of the fibre after its detachment from the matrix (fibre friction, its plastic deformation), for which energy is consumed. Therefore, this is not a process of cement matrix fracture, because other mechanisms affect the increase in the energy consumed during the fracture process. In the fibre concrete fracture process, however, work, defined as the area under the load–deflection curve, is always performed.

#### 2.2.5. Toughness Indices I_5_, I_10_, I_20_

Toughness indices were determined according to ASTM C1018-97 [61] from the load–deflection curve for concrete specimens after 28 days of curing. The load at the limit of proportionality F_LOP_ was calculated according to EN 14651 [57]. For the F_LOP_ load values, (F_LOP_ corresponds to the highest load for the CMOD range from 0 to 50 µm), the σ_LOP_ deflection was determined, followed by the 3 × σ_LOP_, 5.5 × σ_LOP_, and 10.5 × σ_LOP_ deflections, and the loads corresponding to these deflections. The region under the defined areas in the load–deflection curve was then determined and the indices I_5_, I_10_, and I_20_ were calculated. The test result is the average of the eight specimens tested for each concrete mix with MSFs and RTSFs. The difference between the fracture toughness indicator tests and the work of a fracture is that the work of a fracture is determined as the total area under the load–deflection curve, up to 3.02 mm deflection for each sample. The fracture toughness indicators were in turn determined as the ratio of the 3 × σ_LOP_, 5.5 × σ_LOP,_ and 10.5 × σ_LOP_ areas to the area of the deflection at the limit of proportionality σ_LOP._ That is why the fracture strength indicators determine the material’s fracture characteristics only at the beginning of the sample’s cracking. 

#### 2.2.6. Microscopic Analysis

Microscopic analysis was performed in order to observe a fibre–grout contact boundary and the adhesion of the fibre to the concrete matrix. Concrete fractures with MSFs and RTSFs were observed after 28 days of curing. The test was performed using a ZEISS Sigma 500 VP scanning microscope.

#### 2.2.7. Fibre Distribution Imaging with Computer Tomography

The fibre distribution in concrete was analysed on cubic samples with 100 mm × 100 mm × 100 mm side size, cut out from 100 mm × 100 mm × 500 mm concrete beams. Tests were made from the same batch as for three-point fracture tests, but in different forms. The samples contained 10, 20, 30, and 40 kg/m^3^ of RTSFs and MSFs. The test was performed to observe the distribution of recycled tyre steel fibres in cured concrete and check if the fibres were uniformly distributed in the concrete mix during mixing. The test was conducted using General Electric (GE V|TOME|X M300) computer tomography, rendering 3D images of the fibre distribution in cubic samples. 

## 3. Results

### 3.1. Consistency

**Figure 4:**
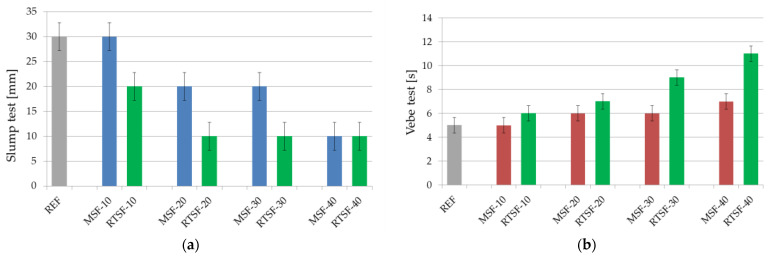
The effect of RTSFs and MSFs on the consistency of the concrete mix: (**a**) slump test, (**b**) Vebe test.

Figure 4 shows the effect of RTSFs and MSFs on the consistency of the concrete mix. The analysis of the rheological properties of fibre concrete is vital because of the significant effect of fibre addition on the reduction in the consistency of concrete mixtures. The results were analysed by comparing concrete mixtures with the same MSF and RTSF mass content.

The results show that as the fibre content increased, both MSFs and RTSFs reduced the consistency of the mix. A higher reduction in consistency was shown by recycled fibres from tyres than standard steel fibres, but the decrease was not significant enough to conclude that there was a technological issue in this scope. One problem would be the inability to test the consistency by any method (Vebe or slump test). A chemical admixture is always added to industrial fibre concrete to improve its consistency. In this case, the reference concrete did not assume an addition of chemical admixture in order to determine the influence of only the fibres’ consistency.

Concrete mix containing RTSFs was homogenous and cohesive even with the addition of up to 40 kg/m^3^ of fibres. The refining process of RTSFs cleans their surfaces of the residues of rubber and textile wastes, which helps in the proper homogenization of the concrete mix. To prove the proper distribution of fibres in the cement matrix, the CT (computer tomography) scanning tests were performed (see Section 3.6.). 

### 3.2. Residual Flexural Tensile Strength

The residual flexural tensile strength test is a standard measure used to evaluate the effectiveness of fibre reinforcement in concrete. When the cement matrix cracks, the crack is the place where fibres and concrete interact as the load and the CMOD increase in a constant and uniform fashion. The determined residual strength is used to measure the effectiveness of reinforcement at specific crack opening values. 

**Figure 5:**
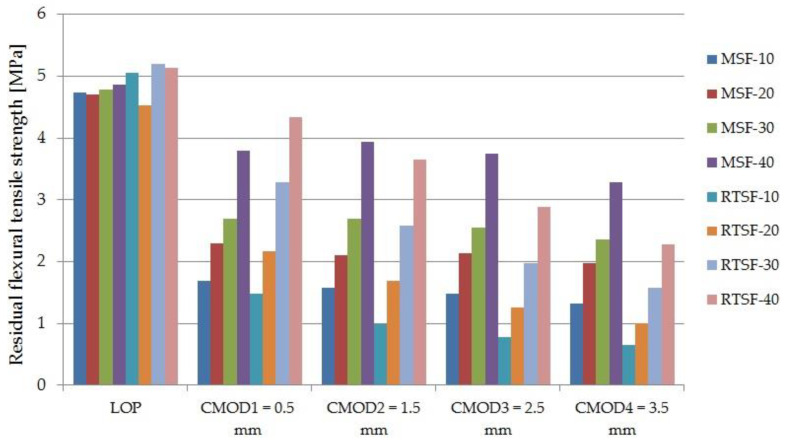
Residual flexural tensile strength test (LOP—limit of proportionality).

**Figure 6:**
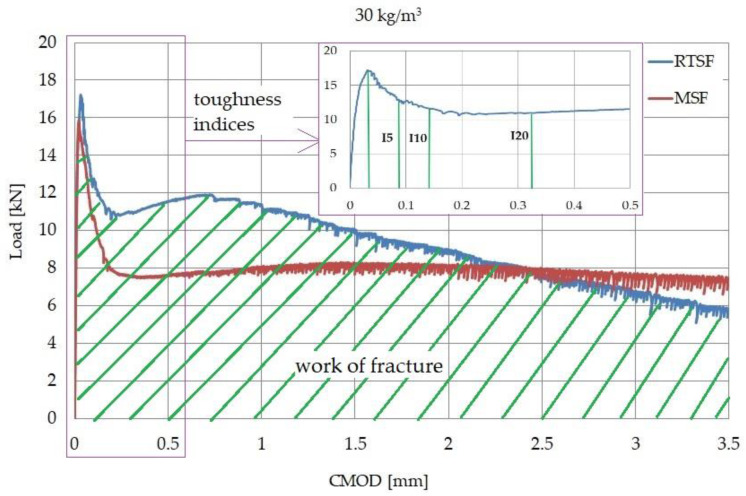
Examples of load–CMOD curve for concrete with 30 kg/m^3^ of the fibres.

**Figure 7:**
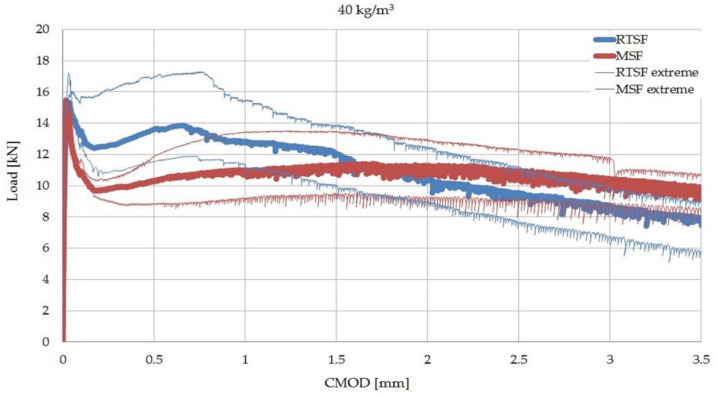
Load–CMOD curves for concrete with with 40 kg/m^3^ of the fibres. The thick lines in the middle show the sample with the most characteristic post-cracking behaviour. The thin lines represent the samples with extreme values from eight tested samples.

Figure 5 shows the results of the residual flexural tensile strength test for types of concrete containing 10, 20, and 30 kg/m^3^ of MSFs and RTSFs, while Figure 6 and Figure 7 present the comparison of the load–CMOD curves for examples of concrete samples containing 30 and 40 kg/m^3^ of the fibres. The data presented in this study are available in the Supplementary Material.

The analysis of the results showed that, compared to manufactured steel fibres, the addition of recycled tyre steel fibres slightly increased the flexural tensile strength at the limit of proportionality. This relationship was observed especially for the fibre contents of 30 and 40 kg/m^3^.

The results of residual flexural tensile strength and the results of the process of fracture and load transfer by the fibres varied between RTSFs and MSFs (Figure 6 and Figure 7). The load–CMOD curve for concrete with MSFs after the cement matrix fracture was constant and uniform. For concrete with RTSFs, however, the load–CMOD curve displayed a characteristic upward trend immediately after the concrete cracks. The effect of more effective RTSF reinforcement compared to MSF reinforcement can be seen from the moment of fracture for the CMOD value reaching approx. 1.3 mm (up to 2.3 mm for some samples).

The analysis of the flexural tensile strength test results for specific CMOD values (0.5, 1.5, 2.5, 3.5 mm) indicated that at 10 and 20 kg/m^3^ of the fibres, RTSFs had a lower residual strength than MSFs. At 30 and 40 kg/m^3^ and a CMOD of 0.5, the concrete with purified RTSFs showed higher residual strength values than the concrete with the same amount of MSFs, which confirms the effectiveness of RTSF reinforcement at higher doses. At the same fibre content and a CMOD of 1.5 mm, the residual strength was the same for concrete with RTSFs and MSFs, while at CMOD of 2.5 and 3.5 mm, the residual strength for concrete with MSFs was higher than that for concrete with RTSFs. The same observations can be made in Figure 6 and Figure 7, showing the higher strengths of concrete containing RTSFs for the CMOD of ca. 1.2–1.5 mm. In the case of a lower dose of fibres, the effect of strengthening the mortar in concrete strongly depends on an effective number of fibres in the cross-sectional area. These recycled fibers are thinner than MSFs, and therefore they are much easier to deform. Therefore, at higher deformations, the number of fibres with a “reach” region of samples on both sides of the crack, comparable to their length, is much smaller than for stiff (due to their diameters) MSFs. At the same time, the number of total fibres in the cross-sectional area is much greater for RTSFs than MSFs, and therefore the effect of their action is especially noticeable at small deformations. Therefore, this value also depends on the dose of fibres in the concrete, as the effect of the number of fibers begins to dominate. To sum up, it can be concluded that the higher the amount of treated recycled tyre steel fibres, the better the effectiveness of concrete reinforcement.

### 3.3. Work of Fracture

While residual flexural tensile strength describes the mechanical properties of fibre concrete for specific fixed CMOD values, work of fracture describes the entire fracture process of cement composites containing fibres, and serves as a measure of fracture resistance and the effectiveness of concrete reinforcement by a given fibre. The work of fracture was calculated based on the measurement of the area under the load (kN)–deflection (mm) curve from the moment of the application of a load to the deflection of 3.02 mm for all analysed types of concrete with MSFs and RTSFs. 

**Figure 8:**
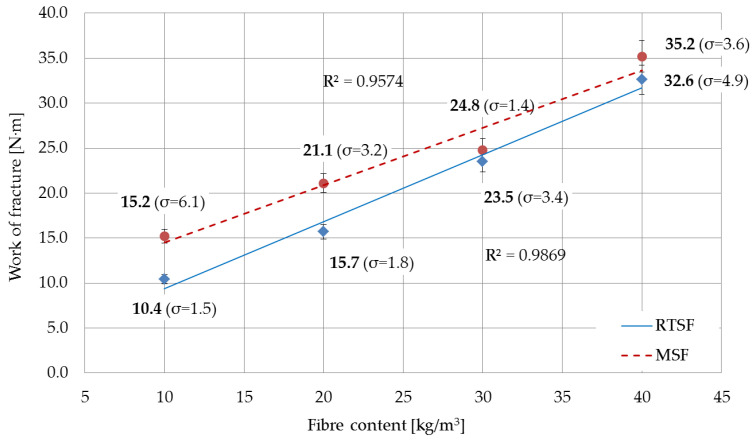
Work of fracture from the moment of applying a force to the deflection of 3.02 mm (σ—standard deviation).

Figure 8 shows the average results from the eight test samples for each amount of MSFs and RTSFs.

The figure shows that the work of the fracture, i.e., the resistance to brittle fracture, for both concrete with MSFs and concrete with RTSFs increased as the fibre content in the concrete increased; however, this correlation was slightly weaker for MSFs (correlation coefficient R^2^ = 0.9574) than for RTSFs (R^2^ = 0.9869). An analysis of the results indicates that at 10 kg/m^3^ and 20 kg/m^3^, concrete with MSFs had higher levels of fractures than concrete with RTSFs, by 32 and 28%, respectively. At 30 kg/m^3^, however, the work of fracture for concrete with MSFs was only 5% higher, while for 40 kg/m^3^_,_ it was 7% higher than that of concrete with RTSFs. Considering the material’s characteristics, the uncertainty, and the standard deviation, it may be concluded that work of fracture, i.e., the fracture resistance, for both comparable composites with 30 and 40 kg/m^3^ of fibres was similar. No papers that defined fracture resistance using the measured work of fracture for concrete containing recycled tyre steel fibres were identified as part of the literature analysis.

These findings can also be seen in Figure 6 and Figure 7, where the fracture process is completely different for both types of concrete. For concrete with RTSFs, the so-called “hump” can be seen on the load–CMOD curve after the cement matrix fractures. This hump is associated with effective fibre reinforcement right after the cement matrix fractures, because fibre reinforcement begins to be effective.

### 3.4. Toughness Indices

**Figure 9:**
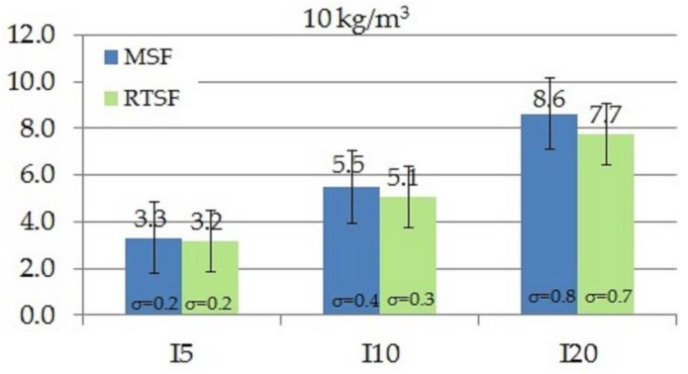
Comparison of fracture toughness indices I_5_, I_10_, I_20_ of concrete with 10 kg/m^3^ MSFs and RTSFs (σ—standard deviation).

**Figure 10:**
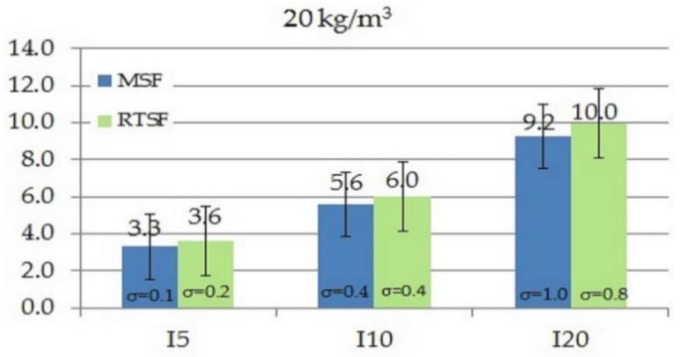
Comparison of fracture toughness indices I_5_, I_10_, I_20_ of concrete with 20 kg/m^3^ MSFs and RTSFs (σ—standard deviation).

**Figure 11:**
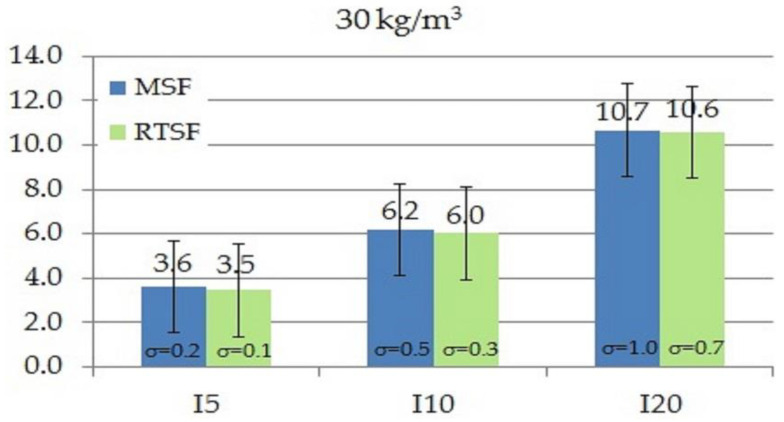
Comparison of fracture toughness indices I_5_, I_10_, I_20_ of concrete with 30 kg/m^3^ MSFs and RTSFs (σ—standard deviation).

**Figure 12:**
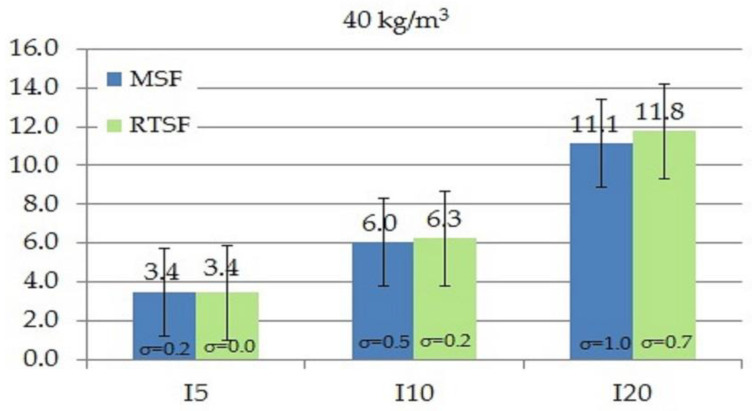
Comparison of fracture toughness indices I_5_, I_10_, I_20_ of concrete with 40 kg/m^3^ MSFs and RTSFs (σ—standard deviation).

As in the case of the work of fracture, toughness indices I_5_, I_10_ and I_20_ tested according to ASTM C1018-97 [61] also define the area under the curve as flexural toughness. The difference is that work of fracture determines fracture resistance from the moment of applying a force to the deflection of 3.02, whereas the indices I_5_, I_10_, and I_20_ are calculated for areas specified for deflections 3, 5.5, and 10.5 times higher, respectively, compared to the area for the deflection under which the first crack occurred. Figure 9, Figure 10, Figure 11 and Figure 12 show the results of comparative tests of the toughness indices for concrete with MSFs and RTSFs.

The analysis of the I_5_, I_10_, I_20_ fracture toughness indices reveals that concrete containing 10 kg/m^3^ of MSFs had higher toughness indices than concrete containing RTSFs. However, at the higher content of RTSFs, the fracture toughness indices reached equivalent or higher values than MSFs. A significant difference can be observed for the 40 kg/m^3^ content, for which the I_10_ and I_20_ fracture toughness indices were higher for RTSF concrete than for the same amount of MSFs. For the RTSF content of >30 kg/m^3^, the reinforcement effectiveness was higher than for the equivalent content of MSFs. 

Considering the material’s characteristics, the uncertainty, and the standard deviation, it can be concluded that the toughness index I_5_ was at the same level (values of 3.2–3.6) for each RTSF and MSF content; this was similar for the I_10_ index (values of 5.1–6.2). For the I_20_ index, a slight increase in the value was observed with the increase in the amount of fibres, both for RTSFs and MSFs.

### 3.5. Microscopic Analysis

**Figure 13:**
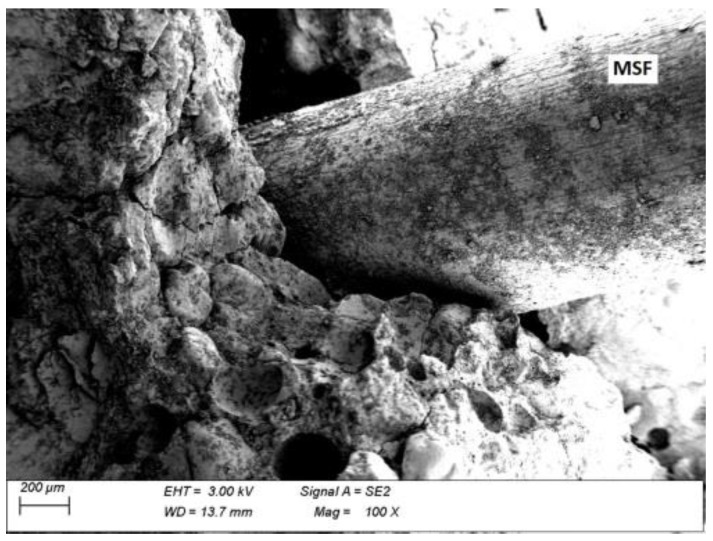
Fracture of concrete with MSFs.

**Figure 14:**
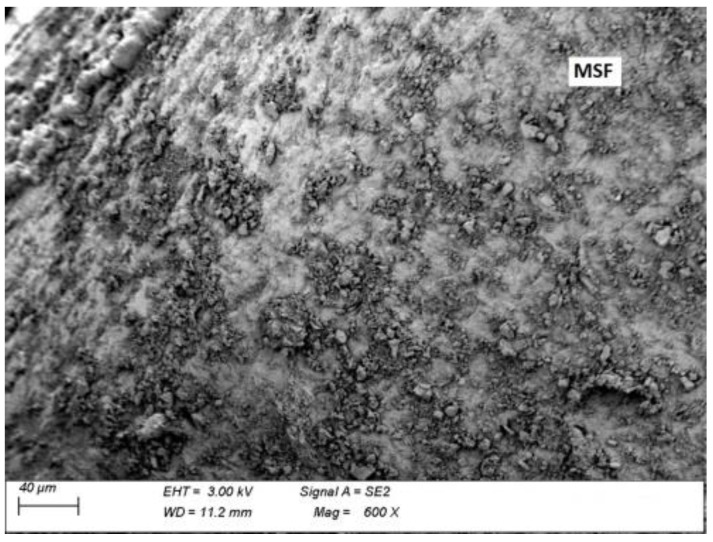
Manufactured steel fibres (MSFs) covered with cement hydration products.

**Figure 15:**
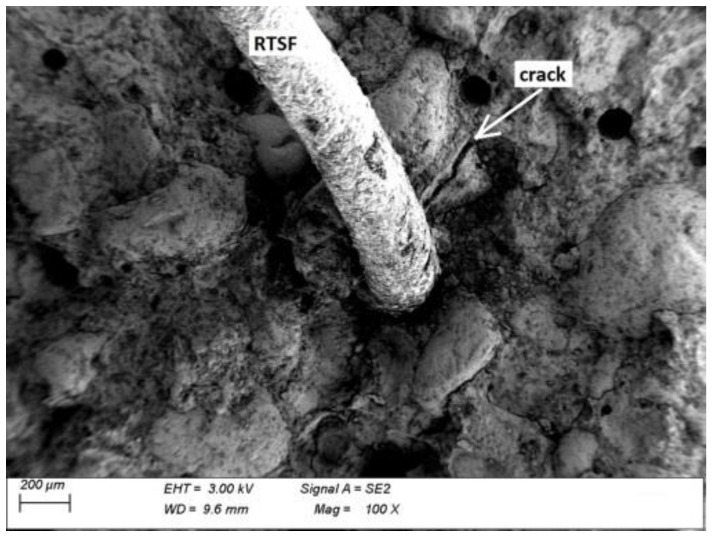
Fracture of concrete with RTSFs.

**Figure 16:**
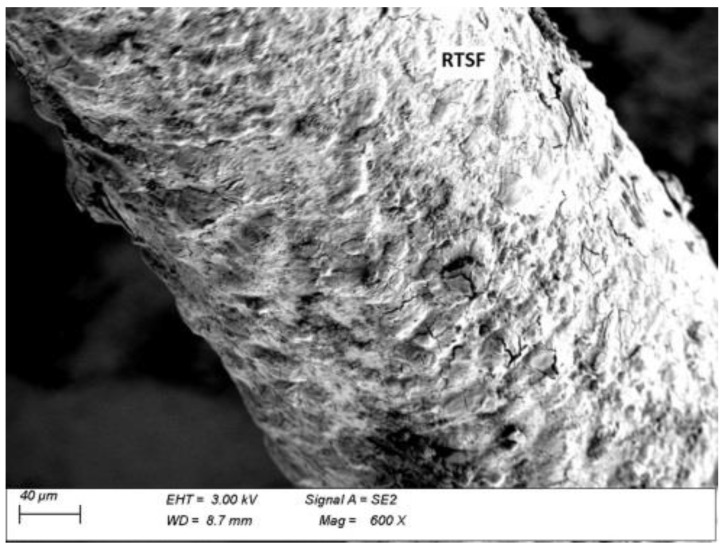
Recycled tyre steel fibres (RTSFs) covered with cement hydration products.

Microscopic analysis was performed in order to observe a fibre–grout contact boundary in concrete fractures. This observation is used to assess the extent to which the fibres anchor and adhere to the grout and the quality of the fibre surface, which affect the effectiveness of fibre reinforcement. Figure 13 and Figure 14 show microscopic images of fractures of concrete with MSFs, while Figure 15 and Figure 16 present fractures of concrete with RTSFs.

The analysis of the microscopic images of concrete fractures reveals that both MSFs and RTSFs were anchored to the grout well, reflecting their good adhesion. The additional process of purifying RTSFs of rubber and textile impurities causes them to have a more developed surface and thus good adhesion to the cement matrix. The fibre–grout contact boundary was proper, compact, and nonporous in both cases. Cement hydration products can be seen on the surface of the MSFs and RTSFs. Additionally, Figure 15 reveals a microcrack which stopped at the fibre–grout contact boundary, i.e., the crack-bridging effect produced by recycled tyre steel fibres.

The microscopic analysis serves as a confirmation and justification of the mechanical test results, pointing to the purity of recycled tyre steel fibres, their good adhesion to the grout, and their effective reinforcement of the brittle cement matrix. As a result, they can be an alternative to manufactured steel fibres (MSFs).

### 3.6. Fibre Distribution with Computer Tomography Method

**Figure 17:**
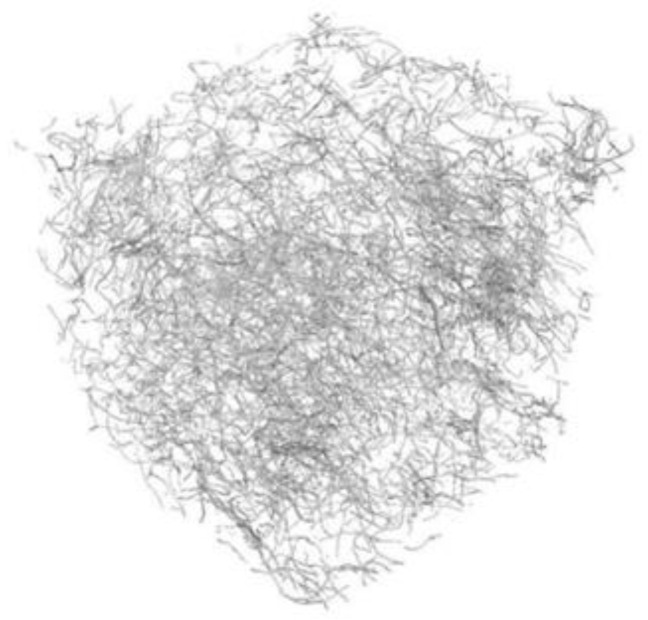
3D computer tomography image with 20 kg/m^3^ RTSFs.

**Figure 18:**
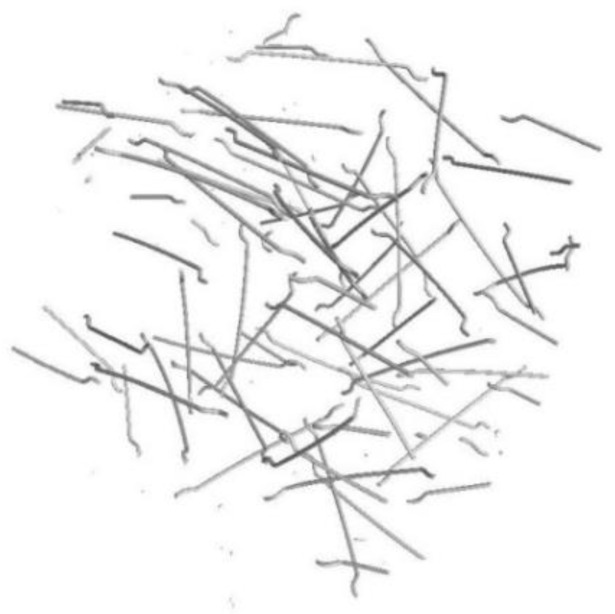
3D computer tomography image with 20 kg/m^3^ MSFs.

**Figure 19:**
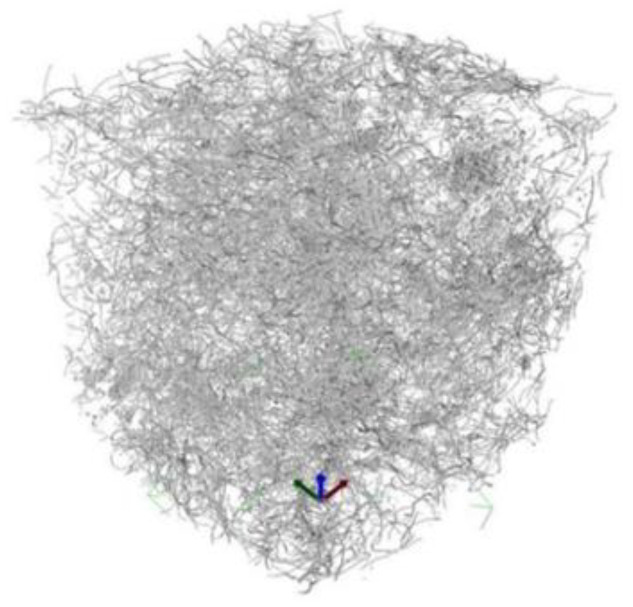
3D computer tomography image with 40 kg/m^3^ RTSFs.

**Figure 20:**
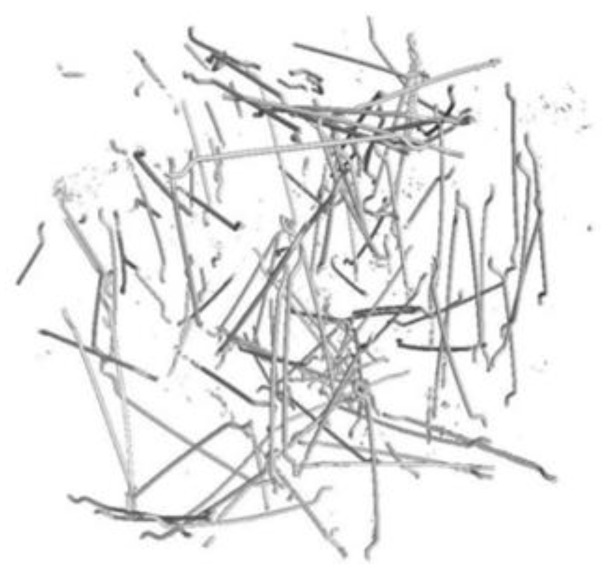
3D computer tomography image with 40 kg/m^3^ MSFs.

Figure 17, Figure 18, Figure 19 and Figure 20 show 3D images of the RTSF and MSF distribution in cubic concrete blocks with 100 mm sides. The images reveal that purified RTSFs were uniformly distributed in the cement matrix for smaller quantities, for example, at 20 kg/m^3^, and at higher contents, such as 40 kg/m^3^. The test was performed because of the concrete reference characteristics according to the EN 14845-1 [55] requirements that were assumed to be free of plasticising admixture. In such reference concrete free of admixture, fibres contribute to a consistency decrease, and there is a risk of not reaching an adequate distribution and good homogenisation of fibres in the concrete. The 3D computer tomography images confirmed the good homogenisation of hybrid RTSFs in concrete, with the fibres characterised by irregular shapes and dimensions.

## 4. Discussion

This article compares MSFs and RTSFs of various shapes and dimensions, which may be debated. In the vast majority of cases, the steel fibres used for concrete are of a similar type to the MSFs used here as a reference. Fibres with a much smaller diameter are available but are very rarely used and are not dedicated to concrete, just like mixtures of fibers with different diameters. Nevertheless, regardless of the type of fibers used, it seems that the most important parameter is the effect of energy absorption during concrete fracture, because it is an advantage for users. In this work, we deliberately used toughness indices to compare the reinforcement effect, because they are universal and by using them, even very different composites can be compared.

This study demonstrated that recycled tyre steel fibres contribute to a slightly higher consistency decrease than the same amount of industrial MSFs, and the higher the fibre content in concrete, the more significant the decrease is.

A reduction in the consistency of fibre concrete with RTSFs has also been shown in previous works [27,38,39], which may be related to the geometric properties of the fibres.

It needs to be emphasised that a reference concrete mix containing no plasticising admixture was used in this study, so the consistency decrease when fibres are added is natural. The RTSFs were uniformly distributed throughout the entire concrete mix volume during mixing, with no clusters formed. Computer tomography of the cubes was performed to observe the fibres’ distribution in the cement matrix, confirming the uniform distribution of the fibres in the concrete. The authors of [62] reached similar conclusions—they used computer tomography to examine cubic samples with RTSFs and discovered the uniform distribution of the fibres and no fibre clusters (“spikes”).

In reference to the residual tensile strength at bending, it was discovered that similar relationships were observed in a previous paper [15], according to which residual flexural tensile strength increases as the RTSF content in concrete increases, and also in the case of mixing a certain amount of RTSFs with MSFs. In [30], concrete types containing 35 kg/m^3^ of RTSFs achieved equivalent flexural strength to concrete types with the same amount of manufactured steel fibres. In [39], however, the strength of types of concrete with RTSFs was lower than that of types of concrete with MSFs, but the paper focused on ultra-high-performance concrete, whose properties cannot be directly compared with the concrete analysed in this paper, because of the higher content of fibres and different specificity of the concrete. 

Determining the work of fracture as a measure of brittle fracture resistance and the effectiveness of concrete fibre reinforcement with RTSFs is an innovative aspect of this paper. No papers were found in the available literature which determined brittle fracture resistance by measuring the levels of fractures for concrete containing RTSFs. The studies on the levels of fractures revealed that when RTSFs are added, the level of fractures rises, and for RTSF contents of 30 and 40 kg/m^3^, this is equivalent to concrete containing MSFs. 

Previous studies on the determination of toughness indices for concrete with RTSFs were also analysed. The authors of [2] analysed the toughness indices for concrete with the same amount of fibres (30 kg /m3), using industrial steel fibers (ISFs) and two kinds of recycled tyre steel fibers: nonpurified RTSFa and purified RTSFs. The results indicated that nonpurified RTSFs have similar toughness indices to industrial fibers (ISFs), while concrete with purified RTSFs showed higher toughness indices than concrete with nonpurified RTSFs and ISFs. The authors of [37] also investigated the toughness indices I_5_, I_10_, and I_20_ for concrete with 30 kg/m^3^ of manufactured fibres and recycled tyre steel fibres. In this paper, the I_5_, I_10_, and I_20_ indices were lower for concrete containing tyre steel fibres than for concrete with manufactured fibres.

To sum up, it can be concluded that, in this paper, the toughness of concrete with additionally treated RTSFs measured on the basis of the toughness indices I_5_, I_10_, and I_20_ is equivalent to and, in some cases, higher than that of concrete with manufactured steel fibres (MSFs). These results may be attributed to the characteristic fracture process of concrete with RTSFs, in which the strength increases on the load–deflection curve after the cement matrix cracks, which is the area where the I_5_, I_10_, and I_20_ indices are defined. 

The good effectiveness of concrete reinforcement with RTSFs can be attributed to the hybrid geometric characteristics of the fibres, which are a mix of fibres of different lengths, diameters, and shapes, allowing the microcrack bridging effect to affect many planes of the cement matrix [43,45,46,47,48,49,50]. It should be stressed that RTSFs were compared with homogeneous manufactured fibres of thicker diameters, identical lengths and, additionally, hook-like shapes, which were regarded as a strong competitor for recycled tyre steel fibres. Besides the geometric properties, the purity of recycled tyre steel fibres also plays a crucial role in the effective reinforcement of cement composites with RTSFs. As supported by numerous scientific papers [15,24,26,34,35,36,37,38,39], rubber and textile contaminants present in untreated tyre steel fibres have a deteriorating effect on reinforcement effectiveness by, for example, creating a continuous barrier on the fibre surface, reducing the adhesion of the fibres to the cement matrix.

All over the world, particular attention is paid to combating growing environmental degradation by limiting the generation of industrial waste, the sustainably managing the resulting waste, saving natural resources, and reducing greenhouse gas emissions that contribute to global warming and the pollution of our planet. Replacing manufactured steel fibres (MSFs) with treated recycled tyre steel fibres (RTSFs) as a concrete reinforcement material has many benefits from an economic and environmental standpoint. The management of large amounts of waste contributes to saving the natural resources used to manufacture steel, which is in line with sustainable construction and the European Green Deal. Further, the additional steel cord treatment technology required to manage RTSFs efficiently has more environmental benefits than the process of manufacturing new steel, which harms the environment and has a very high carbon footprint.

## 5. Conclusions

This paper, which confirms the excellent effectiveness of concrete reinforcement with purified recycled tyre steel fibres and the possibility of replacing manufactured steel fibres with them, is in line with all of the above. 

The following conclusions were formulated based on this study:

- The prerequisite for effective concrete reinforcement with recycled tyre steel fibres is their high purity, with the lowest possible amount of rubber and textile contaminants and steel dust. Such fibres may only be obtained after the purification of the original steel cord obtained from tyres.
- Recycled tyre steel fibres (RTSFs) were added to concrete in the same amount as industrial steel fibres (MSFs) used as scattered reinforcement for concrete, with all tests made and analysed comparatively.
- The geometric characteristics of recycled tyre steel fibres (RTSFs), which are a mix of hybrid fibres of different lengths, diameters, and irregular shapes, are advantageous and contribute to achieving effective microcrack bridging in the cement matrix even better than MSFs.
- The rheological properties of concrete mixes determined by a consistency test revealed that RTSFs had a slightly higher reduction in consistency than MSFs, especially at high fibre contents. Still, this reduction was not high enough to be considered a technological issue in this regard. Concrete mixes, even those containing large amounts of RTSFs and MSFs, were homogeneous in terms of fibre distribution, with no signs of segregation. The fibre distribution examination with computer tomography confirmed the homogenous and uniform distribution of fibres in concrete and no local fibre clusters.
- The properties of residual flexural tensile strength showed that at 10 and 20 kg/m^3^, concrete with RTSFs had lower values than that with MSFs, although concrete with the addition of 30 to 40 kg/m^3^ of RTSFs received higher values by about 20 and 15%, respectively. For a CMOD higher than 0.5–1.5 mm, the residual flexural strength for concrete with RTSFs was lower than that for concrete with MSFs.
- The effectiveness of reinforcing the concrete might be measured by the fracture toughness, described as the work of fracture, referring to the total areas under the load–deflection curve, which is a new approach compared to other studies devoted to the subject matter. In the case of the work of fracture for 10 and 20 kg/m^3^ of the fibres, it was shown that concrete with RTSFs had lower fracture resistance than that with MSFs; however, at 30 and 40 kg/m^3^, the fracture resistance determined by work of fracture was similar for both types of concrete (containing RTSFs and MSFs, respectively). The efficiency of concrete reinforcement as determined by fracture mechanics parameters increases as the content of recycled tyre steel fibres in concrete increases.
- The identified fracture toughness indices, which are the measure of a fracture’s resistance at the initial stage, right after the first fracture, revealed that the higher the RTSF content in the concrete, the higher the fracture’s toughness indices. For the content of 20, 30, and 40 kg/m^3^, concrete with RTSFs revealed equivalent or even higher fracture toughness indicators than concrete containing MSFs.
- The microstructure of fractures of concrete with MSFs and of concrete with RTSFs was proper, with the fibres being anchored to the grout well and cement hydration products being visible on the surface of the fibres. These observations confirmed the good adhesion of the fibres to the grout. The good adhesion of RTSFs to the cement matrix resulted from the additional purifying process, which made the RTSFs’ surfaces more developed.

The parameters describing the fracture mechanics of fibre-reinforced cement composites indicate that recycled tyre steel fibres, if treated well and added to concrete in the right amount, may replace manufactured steel fibres.

## Figures and Tables

**Table 1:** Geometric characteristics of the fibres.

Type of Fibre	Length (mm)		Diameter (mm)		Description
MSF	50.5		1.00		hooked-end steel fibres, homogeneous, round cross-section
RTSF	interval	median	interval	median	hybrid fibres of various lengths and diameters, treated (rubber and textile contaminants < 0.5%)
	7.4–81.6	40.2	0.17–1.34	0.23	

**Table 2:** Tensile strength and elastic modulus test results.

Type of Fibre	Average Diameter of Fibres (mm)	Tensile Strength (MPa)	Elastic Modulus (GPa)
RTSF	0.30 ± 0.08	1418 ± 54	158.8 ± 22.5
	1.34 ± 0.06	1653 ± 40	191.8 ± 31.2
MSF	1.00 ± 0.04	1082 ± 26	201.6 ± 17.8

**Table 3:** Reference concrete composition.

Component	Content (kg/m^3^)
Portland cement CEM I 42.5 R	310
Tap water	171
Natural aggregate 0/2 mm (sand)	432
Natural silica-based aggregate 2/8 mm (gravel)	902
Natural silica-based aggregate 8/16 (gravel)	545

**Table 4:** Tested concretes.

Symbol	Type of Fibre	Fibre Content (kg/m^3^)
REF	reference concrete without fibres	-
RTSF-10	recycled tyre steel fibres, treated, Figure 1b	10
RTSF-20		20
RTSF-30		30
RTSF-40		40
MSF-10	manufactured steel fibres, Figure 1a	10
MSF-20		20
MSF-30		30
MSF-40		40

## Supplementary Materials

The following supporting information can be downloaded at: https://www.mdpi.com/article/10.3390/ma15072444/s1, Figure S1: Load-displacement curves for tyre wires RTSF with a diameter of 0.30 mm (samples 1–10); Figure S2: Load-displacement curves for tyre wires RTSF with a diameter of 0.30 mm (samples 11–20); Figure S3: Load-displacement curves for tyre wires RTSF with a diameter of 0.30 mm (samples 11–20); Figure S4: Load-displacement curves for tyre wires RTSF with a diameter of 1.34 mm (samples 1–10); Figure S5: Load-displacement curves for tyre wires RTSF with a diameter of 1.34 mm (samples 11–20); Figure S6: Load-displacement curves for tyre wires RTSF with a diameter of 1.34 mm (samples 21–30); Table S1: Residual flexural tensile strength.
