# Supplementary material for: Effectiveness of Concrete Reinforcement with Recycled Tyre Steel Fibres
Source: Materials (Basel). 2022 Mar 26;15(7):2444. doi: 10.3390/ma15072444 (PMC9000085; doi:10.3390/ma15072444)
Supplement: Supplementary file 1 [file materials-15-02444-s001.zip › materials-1618985-supplementary.pdf]

Suplementary Material for article

Effectiveness of Concrete Reinforcement with Recycled Tyre Steel Fibres

Agnieszka Michalik, Filip Chyliński, Jan Bobrowicz and Waldemar Pichór

1. Tensile strength test results of RTSF wires

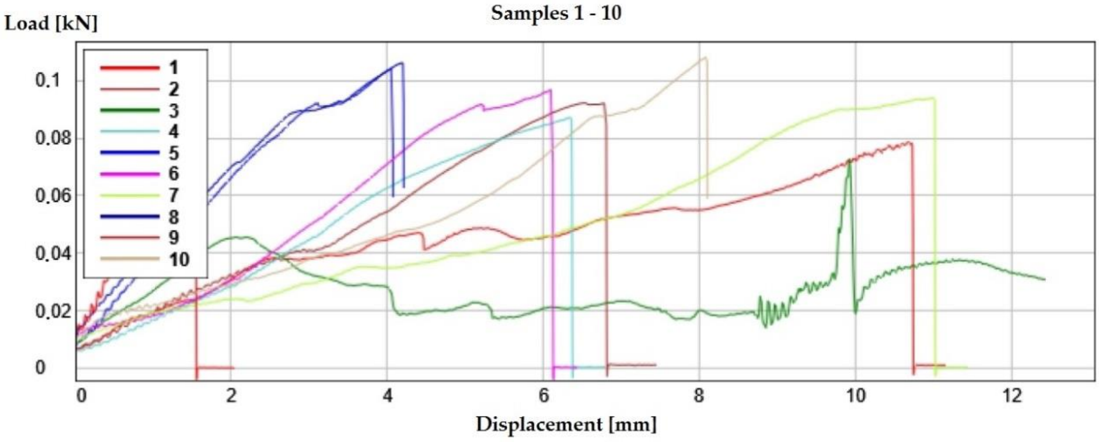

Figure S1. Load-displacement curves for tyre wires RTSF with a diameter of 0.30 mm (samples 1-10).

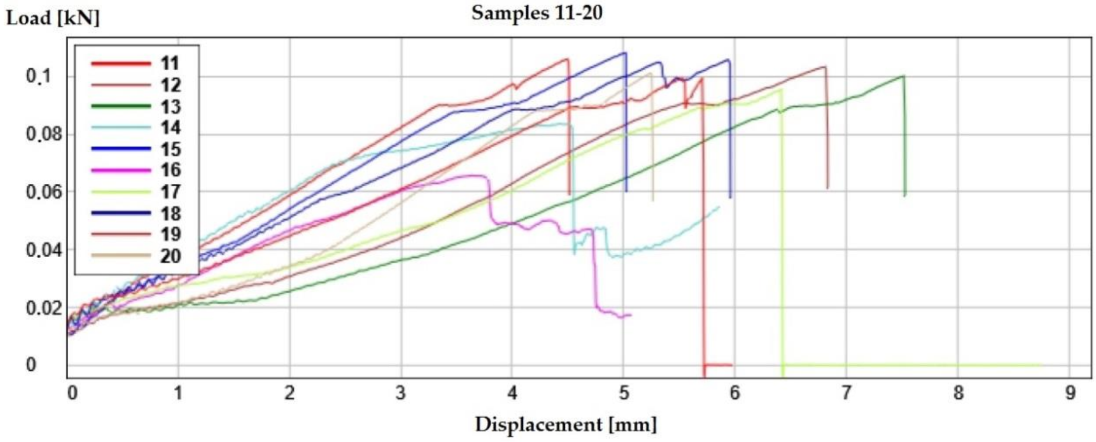

Figure S2. Load-displacement curves for tyre wires RTSF with a diameter of 0.30 mm (samples 11-20).

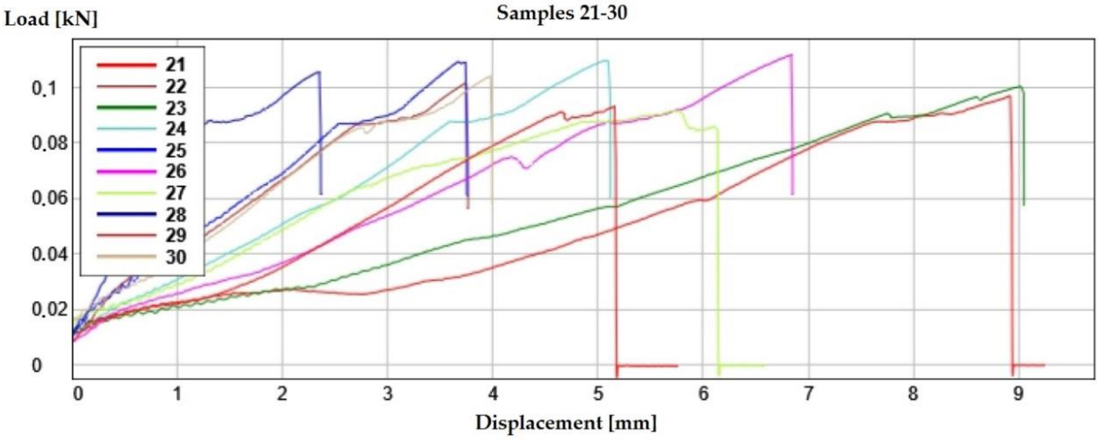

Figure S3. Load-displacement curves for tyre wires RTSF with a diameter of 0.30 mm (samples 21-30).

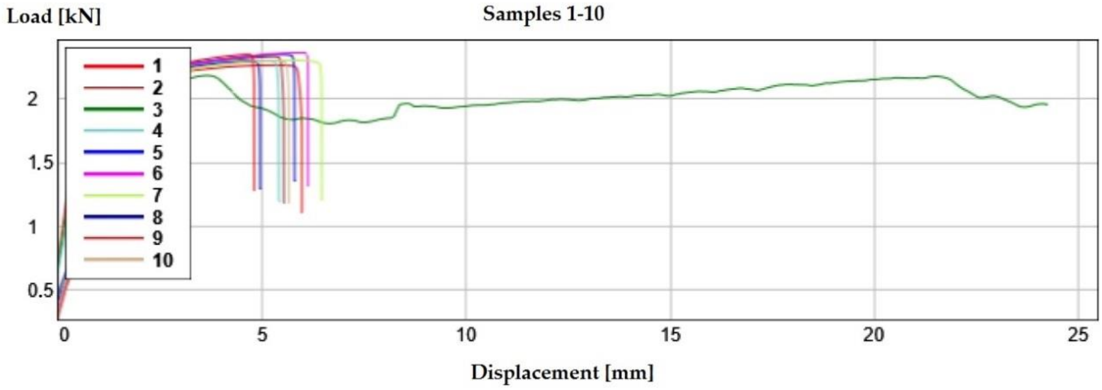

Figure S4. Load-displacement curves for tyre wires RTSF with a diameter of 1.34 mm (samples 1-10).

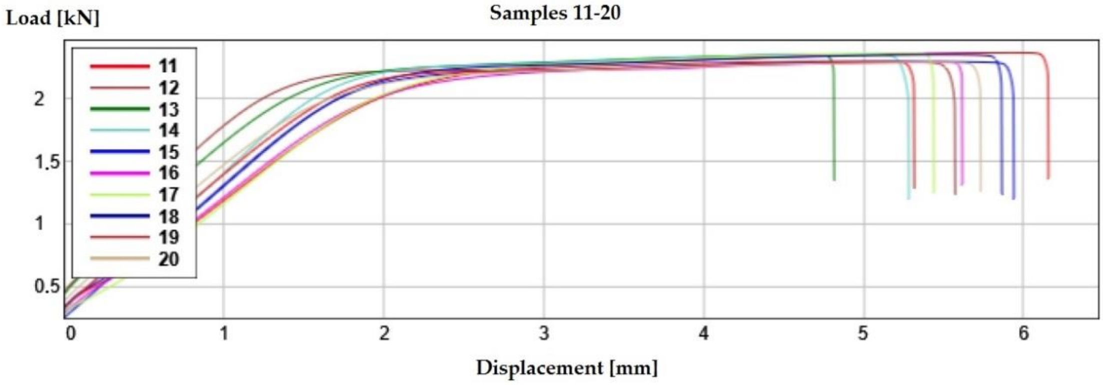

Figure S5. Load-displacement curves for tyre wires RTSF with a diameter of 1.34 mm (samples 11-20).

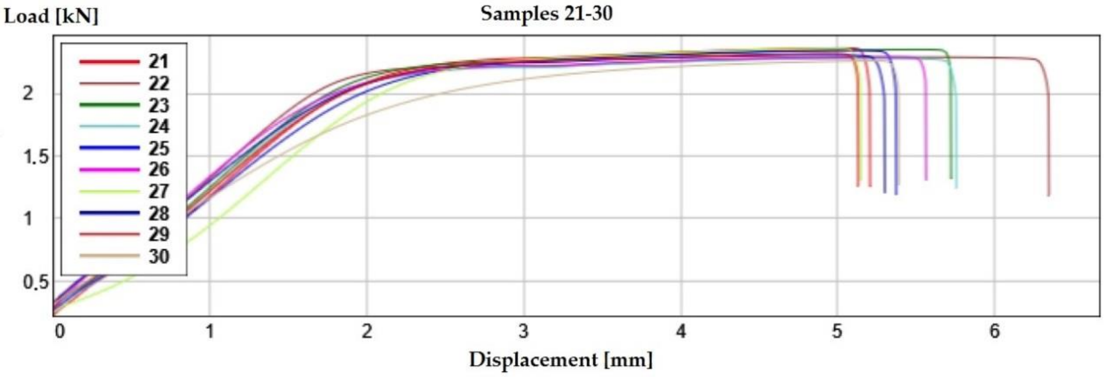

Figure S6. Load-displacement curves for tyre wires RTSF with a diameter of 1.34 mm (samples 21-30).

2. Residual flexural tensile strength of concrete with fibres

Table S1. Residual flexural tensile strength.

Concrete type	Residual flexural tensile strength [MPa]				
	at limit of proportionality (LOP)	residual flexural tensile strength [MPa]			
		$CMOD_1$ = 0.5 mm	$CMOD_2$ = 1.5 mm	$CMOD_3$ = 2.5 mm	$CMOD_4$ = 3.5 mm
MSF-10	4.73	1.69	1.57	1.48	1.32
MSF-20	4.71	2.30	2.10	2.14	1.97
MSF-30	4.79	2.70	2.70	2.55	2.36
MSF-40	4.86	3.79	3.93	3.74	3.28
RTSF-10	5.06	1.48	0.98	0.78	0.65
RTSF-20	4.53	2.16	1.69	1.25	1.01
RTSF-30	5.19	3.28	2.58	1.98	1.57
RTSF-40	5.14	4.33	3.65	2.88	2.28
Uncertainty[1]	±0.34	±0.18	±0.15	±0.16	±0.17

[1] Values of uncertainties are calculated using standard deviation of tested samples set and it also contains factors related with the uncertainties of measuring values e.g. force, crack width, dimensions of beam. Measurement uncertainty is given at an expansion factor of k = 2 and a confidence level of 0.95
